# Comparison of Barricor™ *vs.* lithium heparin tubes for selected routine biochemical analytes and evaluation of post centrifugation stability

**DOI:** 10.11613/BM.2018.020902

**Published:** 2018-04-15

**Authors:** Anne Marie Dupuy, Stéphanie Badiou, Delphine Daubin, Anne Sophie Bargnoux, Chloé Magnan, Kadda Klouche, Jean Paul Cristol

**Affiliations:** 1 Department of Biochemistry, Lapeyronie University Hospital, Montpellier, France; 2 PhyMedExp, INSERM U1046, CNRS UMR 9214, University of Montpellier, Montpellier, France; 3 Intensive Care Medicine Department, Lapeyronie University Hospital, Montpellier, France

**Keywords:** blood specimen collection, plasma, test tube, evaluation

## Abstract

**Introduction:**

Obtaining suitable results unaffected by pre- or postanalytical phases is pivotal for clinical chemistry service. We aimed comparison and stability of nine biochemical analytes after centrifugation using Barricor™ plasma tubes with mechanical separator *vs* standard Vacutainer® lithium heparin tubes.

**Materials and methods:**

We collected samples on six healthy volunteers and nine patients from intensive care units into 6 mL plastic Vacutainer® lithium heparin tubes and 5.5 mL plastic Barricor™ plasma tubes. All tubes were centrifuged within 30 minutes after venipuncture. First, we compared results of nine biochemical analytes from lithium heparin tubes with Barricor™ tubes for each analyte using Passing-Bablok and Bland-Altman analyses. Second, we calculated the difference of analyte concentrations between baseline and time intervals in tubes stored at + 4 °C. Based on the total change limit we calculated the maximum allowable concentrations percentage change from baseline.

**Results:**

The majority of correlation coefficients were close to 0.99 indicating good correlation in the working range. Bland-Altman analyses showed an acceptable concordance for all analytes. In consequence, the Barricor™ tube might be an alternative to regular lithium heparin tube. Stability with this new generation tube is improved for eight analytes (except for aspartate aminotransferase) in comparison with regular lithium heparin tubes.

**Conclusions:**

By using Barricor™ tubes and prompt centrifugation, supplemental analysis or re-analysis for eight analytes including alanine aminotransferase, alkaline phosphatase, C-reactive protein, high sensitivity troponin T, lactate dehydrogenase, NT-pro BNP, potassium and sodium could be performed within 72 h of specimen collection.

## Introduction

Many clinical laboratories for routine analytes have been using plasma or serum separator tubes for several years. The barrier gel allows rapid separation of serum or plasma from blood cells and reduces haemolysis. The gel used in these tubes is relatively inert, however several reports of gel affecting analyte concentrations have been published (*1*, *2*).

Becton, Dickinson and Company (BD) has recently developed the BD Barricor™ plasma blood collection tube, which has a mechanical separator, rather than a gel barrier. According to the manufacturer, the quality of plasma is improved, the centrifugation time is shorter (3 min *vs.* 10 min with BD Vacutainer® lithium heparin tubes) and post centrifugation analyte concentrations are more stable in samples that have been stored in the primary tube. At this time, some studies have been published to verify if these tubes are an effective alternative to gel barrier tubes (*3*-*5*). Nevertheless, these publications concern mainly the correlations between results obtained using different tubes for biochemical parameters, particularly cardiac markers, without stability studies (*3*, *4*). The one study which has investigated different storage conditions relates only to electrolytes (*5*). Given this lack of data, we aimed to compare results for nine biochemical tests from the BD Vacutainer® lithium heparin tubes *vs.* the BD Barricor™ plasma tubes and to determine plasma stability of analytes after centrifugation according to our routine laboratory practice.

## Materials and methods

### Subjects, blood sampling and analysis

Six healthy volunteers (physicians) and nine patients from intensive care units (Lapeyronie Hospital, Montpellier) were included in the study group. Healthy volunteers as well as hospitalized patients were selected to cover both normal and pathological analyte levels. All the samples were obtained in the morning between 8 and 9 am after overnight fasting for healthy volunteers. The study was performed at Lapeyronie Hospital, Montpellier in June 2017, with appropriate ethics approval and with informed consent from all subjects. Two blood samples were drawn from each participant: one was collected into a 6 mL plastic BD Vacutainer® lithium heparin tube and the other into a 5.5 mL plastic BD Barricor™ plasma tube (Becton, Dickinson and Company, Franklin Lakes, NJ, USA). Both types of tubes were transported to the laboratory and centrifuged without automated pre-analytical system within 30 min after venipuncture at 20 °C in a dedicated Heraeus Multifuge X3 FR Centrifuge (Germany) according to the manufacturer’s recommendations (2000 x g for 10 min for lithium heparin tubes; 4000 x g for 3 min for Barricor™ plasma tubes). Thereafter, tubes were loaded on biochemistry analyser defining the time T0. Nine routine chemistry parameters were measured: alanine aminotransferase (ALT), alkaline phosphatase (ALP), aspartate aminotransferase (AST), C-reactive protein (CRP), high sensitivity troponin T (hs-cTnT), lactate dehydrogenase (LD), N-terminal pro brain natriuretic peptide (NT-pro BNP), potassium (K) and sodium (Na). All these biochemical analytes were quantified on a Cobas 8000^©^ system including c701 and c502 module (for biochemical analytes) and e602 module (for immunoassays) from Roche Diagnostics (Meylan, France) using Roche reagent kits and calibrators. After the first analysis (T0), primary tubes were stored at + 4 °C according to our routine daily storage condition and re-analyzed at different time intervals (after 4, 24 and 72 h). The 72 h deadline was chosen because it corresponds to our daily practice. The visual appearance of the two types of tube at each analysis time was monitored.

### Statistical analysis

First, Passing-Bablok regression analysis was used to compare results from lithium heparin tubes with Barricor™ tubes for each analyte. The slopes and the intercepts were considered non-significant when the 1 or 0 values fell within the 95% CI, respectively. Mann Whitney U-tests were used and the level of significance was set at P < 0.05. In addition, the scatter of differences was visualized by means of Bland-Altman plots and the calculated mean bias was reported for each analyte. Further, 95% confidence intervals (mean difference ± 1.96 SD) were reported to judge if biases are acceptable in medical practice. In addition, the bias was calculated for each analyte by the formula: [(average of Barricor results – average of standard lithium heparin results / average of standard lithium heparin results)] x 100. This percentage was compared with the desirable bias available from Ricos *et al.* based on biological variation for nine parameters (*6*).

For each storage time and for each analyte, the absolute difference of each sample (or percentage deviation) against the T0 concentration was calculated for the two types of tubes using the formula: [(Tx - T0_Ref_) / T0_Ref_] x 100. The mean percentage deviation (± SD) of the fifteen donors was calculated for the two types of tube. To assess the stability of analytes, we calculated total change limit (TCL) as described by Oddoze *et al*. by using the formula: [(2.77 x CVa)^2^ + (0.5 x CVb)^2^]^½^ where CVa is analytical imprecision and CVb is within-subject variation (*7*). If mean percentage deviation (± SD) was higher than TCL value calculated for an analyte, then the analyte was considered not stable at + 4 °C for that length of storage. Statistical analyses were performed using XLSTAT® software, version 2016.06.35661 (NY, USA).

## Results

### Results comparison at T0

**Figure 1:**
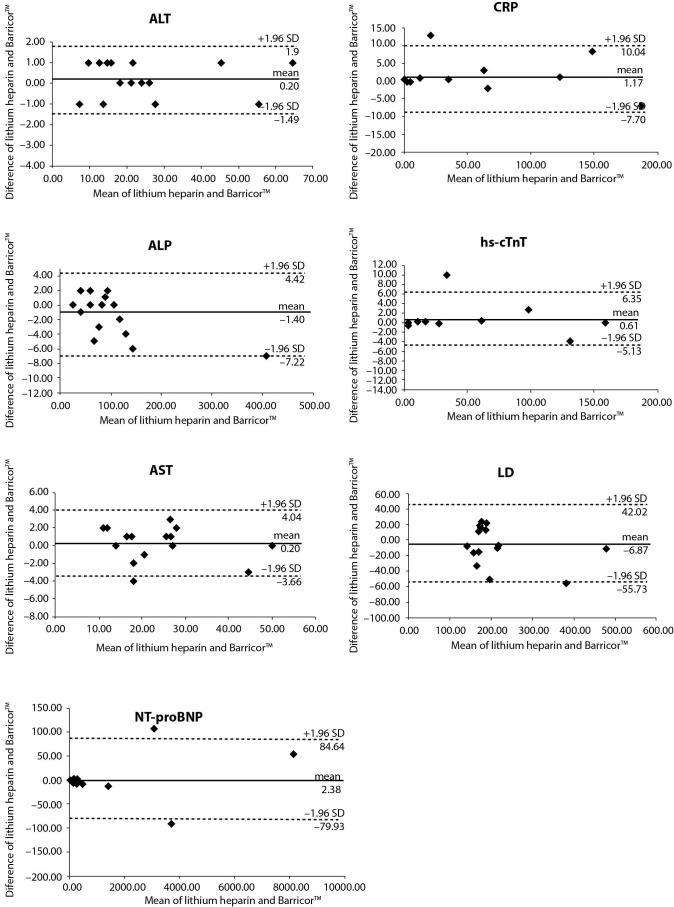
Bland-Altman difference plots for the nine biochemical analytes obtained with BD lithium heparin tubes and BD Barricor™ plasma tubes. The solid line shows the average of the differences obtained and corresponds to the bias between results obtained with two types of tubes. The dashed lines correspond to the lower and upper limits of the confidence interval at 95% (mean ± 1.96 SD) of the difference between results obtained with two types of tubes. ALT - alanine aminotransferase. AST - aspartate aminotransferase. CRP - C-reactive protein. LD - lactate dehydrogenase. ALP - alkaline phosphatase. hs-cTnT - high sensitivity troponin T. NT-proBNP - N-terminal prohormone of brain natriuretic peptide.

Table 1 shows the regression analysis results of the values for the nine analytes obtained from regular lithium heparin tubes *vs* the values from Barricor™ lithium heparin tubes. The majority of correlation coefficients were close to 0.99 (ranging from 0.98 to 1.00). We observed a slopes homogeneity (0.99 to 1.03) as well as intercepts (- 1.5 to 3.74). No difference in the median concentrations for the nine analytes was detected. The concentrations were of the same order of magnitude. However, a slight over-estimation was observed for Na values with Barricor™ tube (the zero value of intercept did not fall within the 95% CI). Overall, Bland-Altman analyses demonstrated an acceptable clinical concordance for all analytes between the two different tubes with a maximum mean difference of - 6.87% (95% CI: 42.02 to -89.23) for LD. All results of the nine biochemical parameters from Barricor™ tube (ALT, ALP, AST, CRP, hs-cTnT, LD, NT-pro BNP, K, and Na) were not statistically different from results of standard lithium heparin tube. For the eight analytes the calculated bias was inside the maximum desirable bias. However, the calculated bias value for Na exceeded the value of desirable bias, although minimally (0.44% *vs.* 0.23%, respectively). The Na values between the two types of tubes were strongly correlated as observed with Passing Bablok regression. According to Bland-Altman plots (Figure 1), only one paired results was outside the limit of agreement.

**Table 1:** Comparison between regular lithium heparin tubes and Barricor™ lithium heparin tubes for nine biochemical analytes

**Analytes**	**Measuring range**	**Slope** **(95% CI)**	**Intercept** **(95% CI)**	**Regular lithium heparin tube**	**Barricor^TM^ tube**	**Mean difference (%)** (95% CI)*	**Bias (%)**	**Desirable bias (%)**	**P**
**ALT, U/L**	7 - 65	1.00 (0.97 to 1.05)	0.00 (- 1.50 to 0.38)	21 (14 - 27)	21 (14 - 27)	0.20 (- 3.50 – 1.89)	- 0.7	11.5	0.933
**ALP, U/L**	26 - 401	1.02 (1.00 to 1.06)	- 1.48 (- 4.7 to 0.00)	82 (58 - 112)	82 (57 - 113)	- 1.40 (- 10.07 - 4.06)	0.9	6.7	0.983
**AST, U/L**	12 - 50	1.03 (0.91 to - 1.16)	- 1.5 (- 393 to - 1.16)	20 (17 - 28)	21 (17 - 27)	0.20 (- 7.176 - 4.06)	- 1.3	6.5	0.819
**CRP, mg/L**	0.3 - 183	0.99 (0.95 to 1.03)	0.00 (- 0.08 to - 0.11)	13.7 (1.7 - 64.7)	12.7 (1.7 - 64.3)	1.17 (- 18.50 - 10.04)	- 3.8	25.5	0.917
**hs-TnT, ng/L**	3 - 159	0.99 (0.97 to 1.03)	0.00 (- 0.09 to 0.08)	11 (3 - 50)	11 (3 - 45)	0.61 (- 11.84 - 6.35)	- 0.8	23.7	0.932
**LD, U/L**	137 - 475	1.01 (0.60 to 1.42)	3.74 (- 84.68 to 75.20)	183 (168 - 204)	178 (165 - 220)	- 6.87 (- 89.23 - 42.02)	3.2	4.3	1.000
**NT-proBNP, ng/L**	30 - 8143	1.00 (0.99 to 1.02)	0.62 (- 2.23 to 4.74)	261 (135 - 1159)	264 (137 - 1170)	2.36 (- 163.54 - 84.64)	0.9	4.7	0.917
**K, mmol/L**	3.4 - 4.9	1.00 (0.92 to 1.14)	0.00 (- 0.6 to - 0.28)	4.0 (3.8 - 4.3)	4.0 (3.8 - 4.3)	0.02 (- 0.29 - 0.16)	- 0.5	1.8	0.630
**Na, mmol/L**	130 - 150	1.00 (0.83 to 1.00)	1.00 (1.00 to 23.66)	139 (138 - 142)	141 (138- 142)	- 0.60 (- 2.92 - 1.18)	0.4	0.2	0.570
ALT - alanine aminotransferase. AST - aspartate aminotransferase. CRP - C-reactive protein. LD - lactate dehydrogenase. ALP -alkaline phosphatase. hs-cTnT - high sensitivity troponin T. NT-proBNP - N-terminal prohormone of brain natriuretic peptide. K – potassium. Na – sodium. CI - confidence intervals. Values of biochemical analytes tested are given as median and interquartile range. P < 0.05 is considered statistically significant. *95% confidence intervals were calculated by the mean difference ± 1.96 SD.									

### Analyte stability over time

Table 2 summarizes the results for all plasma analytes and the mean differences at several storage times at + 4 °C for both regular lithium heparin and Barricor™ lithium heparin primary tubes. Using TCL as the measure of acceptability, the mean difference exceeded the TCL after 4 h for K and LD in lithium heparin tube samples. In contrast, these two analytes were stable for 72 h using Barricor™ plasma tubes. For both types of tube, AST was only stable up to 24 h. After longer storage (measurement at 72 h), AST increased for 11.96% and 12.37%, exceeding the acceptable TCL. Excluding AST, there were only negligible changes in the plasma concentration of all the other analytes up to 72 h for Barricor™ lithium heparin tube samples.

**Table 2:** Stability of plasma biochemical analytes stored over time in primary tubes tested

**BD lithium heparin tubes**					
**Analyte**	**TCL (%)**	**Mean % difference at + 4 °C**			**Acceptable delay**
		**T4h**	**T24h**	**T72h**	
ALT, U/L	± 21.75	6.02 (6.74)	4.04 (9.86)	1.40 (6.34)	72 h
ALP, U/L	± 14.34	- 0.47 (1.78)	- 0.86 (1.87)	- 1.13 (2.49)	72 h
AST, U/L	± 10.83	7.45 (7.03)	4.40 (8.27)	11.96 (7.60)	24 h
CRP, mg/L	± 38.72	- 4.96 (12.46)	- 1.22 (12.70)	- 3.97 (12.70)	72 h
hs-TnT, ng/L	± 9.37	- 0.01 (5.15)	- 0.77 (2.45)	5.78 (20.20)	72 h
LD, U/L	± 8.82	7.84 (8.13)	10.19 (11.06)	15.98 (16.08)	4 h
NT-proBNP, ng/L	± 18.35	0.52 (2.00)	2.25 (3.10)	- 6.37 (7.28)	72 h
K, mmol/L	± 6.21	0.51 (1.07)	12.29 (7.12)	53.39 (26.72)	4 h
Na, mmol/L	± 3.64	0.72 (0.47)	1.25 (0.88)	0.34 (0.53)	72 h
**BD Barricor lithium heparin tubes**					
**Analyte**	**TCL (%)**	**Mean % difference at + 4 °C**			**Acceptable delay**
		**T4h**	**T24h**	**T72h**	
ALT, U/L	± 21.75	2.39 (8.57)	- 1.59 (7.97)	1.98 (10.64)	72 h
ALP, U/L	± 14.34	- 1.42 (2.50)	- 0.93 (2.90)	- 1.74 (3.36)	72 h
AST, U/L	± 10.83	4.36 (11.14)	5.60 (11.05)	12.37 (14.07)	24 h
CRP, mg/L	± 38.72	- 1.73 (2.96)	2.14 (3.52)	- 0.49 (5.53)	72 h
hs-TnT, ng/L	± 9.37	0.72 (9.03)	2.27 (9.28)	6.00 (17.13)	72 h
LD, U/L	± 8.82	2.96 (3.96)	4.67 (4.13)	8.31 (10.22)	72 h
NT-proBNP, ng/L	± 18.35	- 0.78 (2.27)	0.33 (3.22)	- 7.72 (7.32)	72 h
K, mmol/L	± 6.21	1.16 (1.30)	1.88 (1.18)	4.90 (1.92)	72 h
Na, mmol/L	± 3.64	0.00 (0.53)	0.52 (0.50)	1.00 (0.45)	72 h
ALT - alanine aminotransferase. AST - aspartate aminotransferase. CRP - C-reactive protein. LD - lactate dehydrogenase. ALP -alkaline phosphatase. hs-cTnT - high sensitivity troponin T. NT-proBNP - N-terminal prohormone of brain natriuretic peptide. K – potassium. Na – sodium. TCL - total change limit. SD – standard deviation. T4h, T24h, T72h – storage times in hours. Mean % differences are presented as mean (standard deviation).					

## Discussion

The correlation and the agreement in the working range of Barricor™ lithium heparin tube results with those of the regular lithium heparin tube were acceptable as seen by the regression and the Bland-Altman analysis with the majority of paired data within the agreement limits. However, Na concentrations in Barricor™ plasma tube tend to be higher than Na in regular lithium heparin tube. Since the analytical method is the same for both types of tubes, this might be explained with different acceleration during centrifugation process between kinds of tubes. The biological variation for sodium is very small (0.23%) and the calculated bias is close to the desirable bias (0.44%); for these reasons, we considered that the observed differences were not clinically relevant and results remained in normal range. Those data indicate that the use of Barricor™ tubes did not affect the concentrations of the nine analytes suggesting that Barricor™ tubes may be used interchangeably with regular lithium heparin tubes.

Further, we reported that concentrations for eight analytes remained stable up to 72 h in Barricor™ plasma tube, while AST activity increased significantly after 24 h whatever the type of tubes. Clearly, the Barricor™ plasma tubes improve post centrifugation stability in comparison with BD Vacutainer® lithium heparin, particularly for parameters sensible such as K and LD.

This study confirms the manufacturer’s claims concerning the visual quality of plasma, the shorter centrifugation time, which does not affect the concentrations of analytes, and the longer stability of analytes in samples stored post centrifugation in the primary Barricor™ lithium heparin tube. Therefore, supplemental analysis or re-analysis could be performed within 72 h of specimen collection for eight analytes (except AST) with Barricor™ lithium heparin tube samples.

The main limitation of our study is the number of samples available, but the recruitment of pathological subjects allowed the evaluation of values covering a large measurement range. The small number of samples evaluated could also explain the differences observed between the 2 types of tubes for the results of Na. In previous studies, results obtained from Barricor™ tubes were compared to plasma separator tube (PST) or serum with (SST) or without gel barrier (*3*-*6*). More recently, concentrations of analytes from PST, SST and Barricor™ tubes were compared according to different centrifugation conditions (*8*). In this context, our study provides additional data since it was performed with analytes not previously investigated with Barricor™ tube in comparison with lithium heparin tube and for which post centrifugation stability was evaluated. In addition, further work would be required to evaluate these new tubes on other analytical platforms to assess if the mechanical separator affects the concentrations of other biochemical analytes and particularly the concentrations of therapeutic drugs as previously described for BD SST tubes (*9*, *10*). The improvement of analytes stability is a capital data in regard with increasing trends in the field of biochemistry towards the consolidation of testing laboratories using platforms that are more and more automated. By using Barricor™ lithium heparin tubes, which offer increased post centrifugation stability of analytes, we foresee that, if the centrifugation is carried out quickly after sampling and the tubes are stored at + 4 °C, primary tubes could be transported to the central laboratory and analysed at least up to 24 h post centrifugation.

In conclusion, at initial measure, the two different tube types are interchangeable for the nine analytes investigated on Roche platform. Our data demonstrated that the great advantage of these tubes is the stability of analytes after centrifugation during 72 h, allowing re-analyses or additional analyses if necessary for eight out of 9 measured analytes. Except for AST, analytes including ALT, ALP, CRP, hs-cTnT, LDH, NT-pro BNP, K and Na were unaffected when stored at + 4 °C for up 72 h after centrifugation.
